# Supplementary material for: Biocrusts buffer against the accumulation of soil metallic nutrients induced by warming and rainfall reduction
Source: Commun Biol. 2020 Jun 24;3:325. doi: 10.1038/s42003-020-1054-6 (PMC7314843; doi:10.1038/s42003-020-1054-6)
Supplement: Supplementary file 1 — Supplementary Information [file 42003_2020_1054_MOESM1_ESM.pdf]

Supplementary material to "**Biocrusts buffer against the accumulation of soil metallic nutrients induced by warming and rainfall reduction**" by Moreno-Jiménez et al.

Supplementary Figures 1, 2, 3, 4, 5 and 6.

Supplementary Tables 1, 2, 3 and 4.

Supplementary Figure 1. Total K (a), Mg (c) Fe (e) and Zn (f) and available K (b), Mg (d), Fe (f) and Zn (h) in soils (mg kg^{-1}) at the beginning of the experiment (2010) in low and high biocrust cover plots (box and whisker plots, n=40).

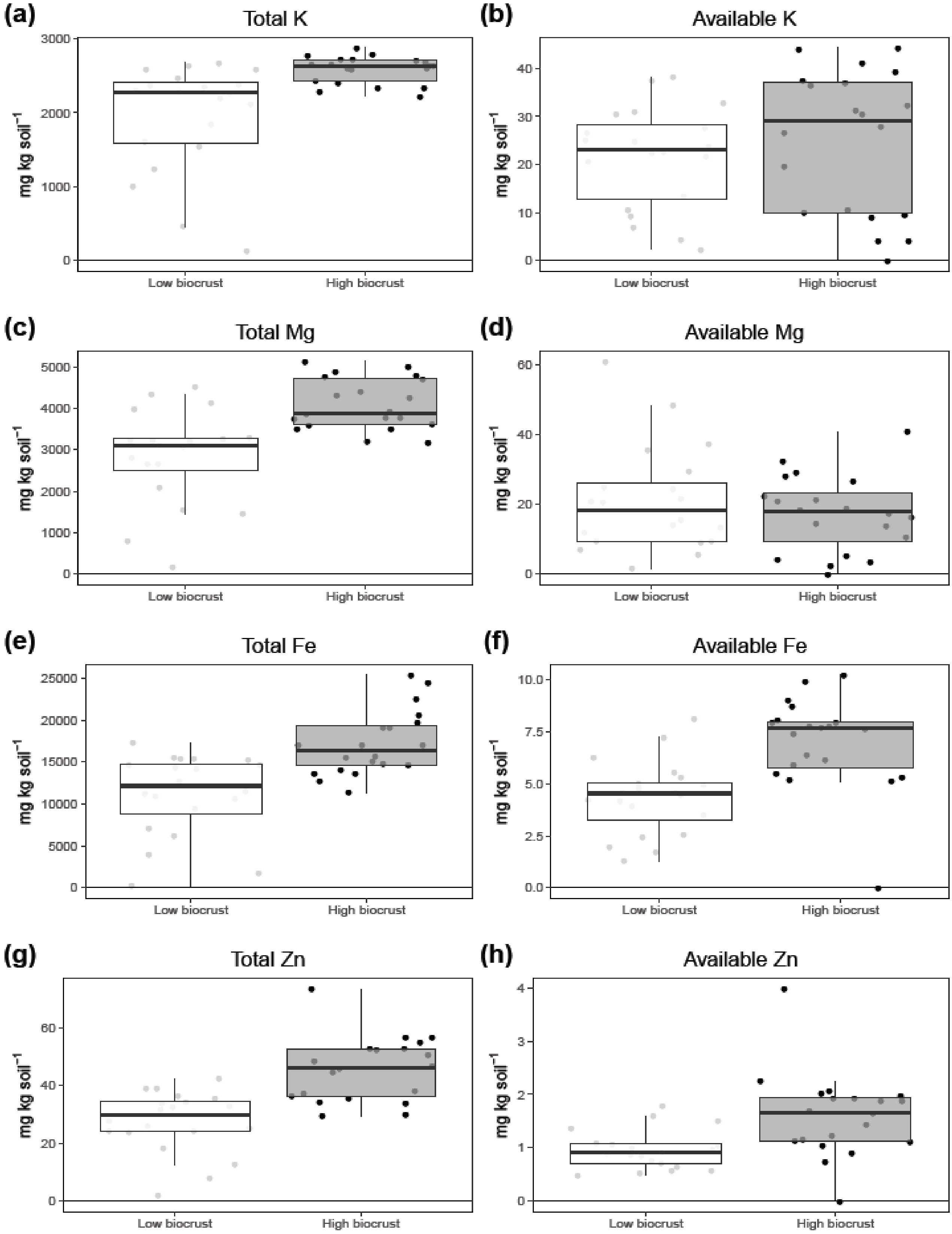

Supplementary Figure 2 Organic C (a), soil pH (b), total Na (c), Cu (e) and Mn (g) and available Na (d), Cu (f) and Mn (h) in soils (mg kg^{-1}) at the beginning of the experiment (2010) in low and high biocrust cover plots (box and whisker plots, n=40).

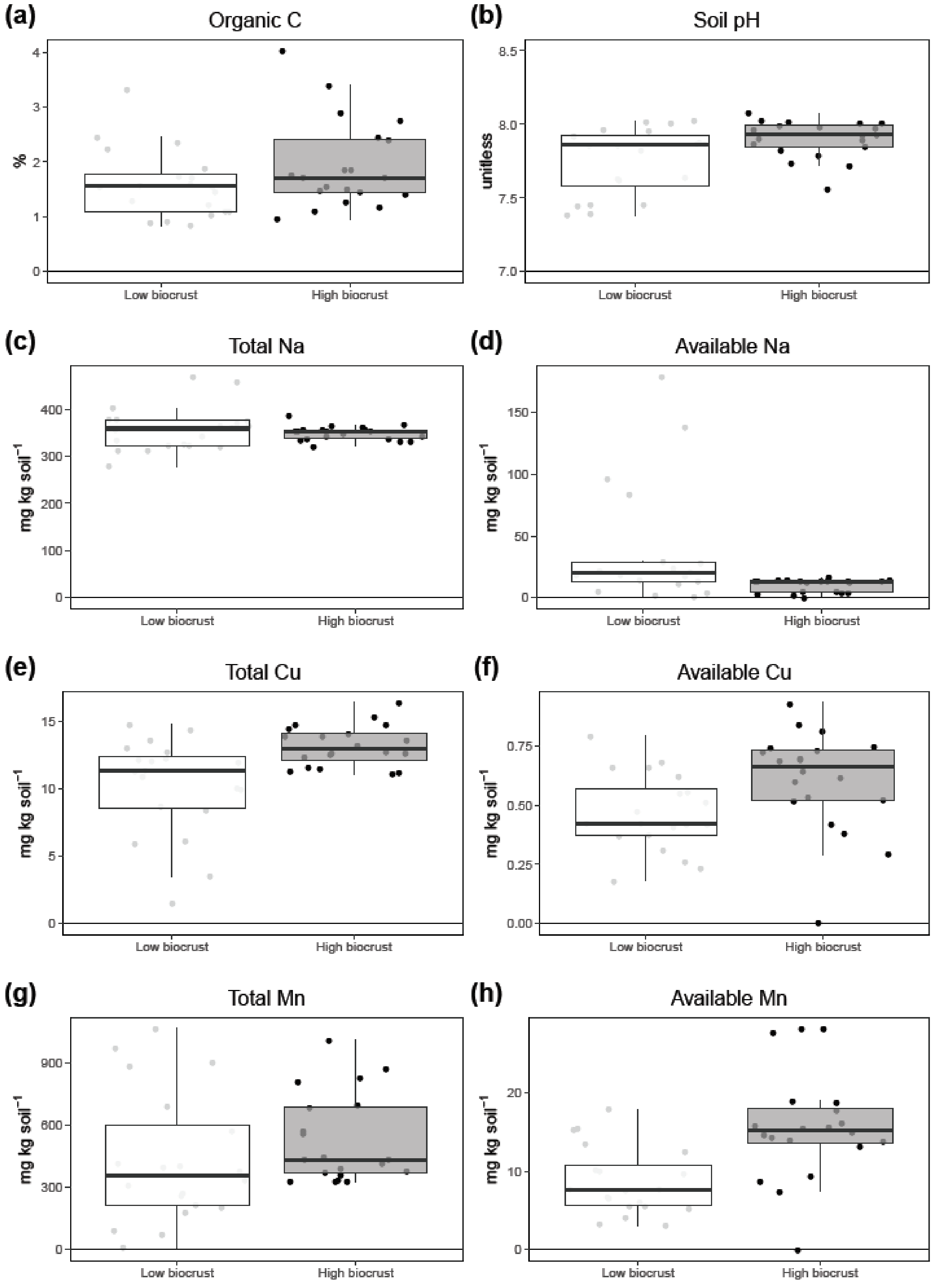

Supplementary Figure 3. Organic C (a), pH (b), total Na (c), Cu (e) and Mn (g) and available Na (d), Cu (f) and Mn (h) in soils (mg kg^{-1}) under the different biocrust cover (low biocrst vs. high biocrust), warming and rainfall exclusion levels evaluated seven years after the beginning of the experiment: control (Amb.), rainfall exclusion (Rain), warming (Warm) and rainfall exclusion and warming (RxW) (box and whisker plots, n=5).

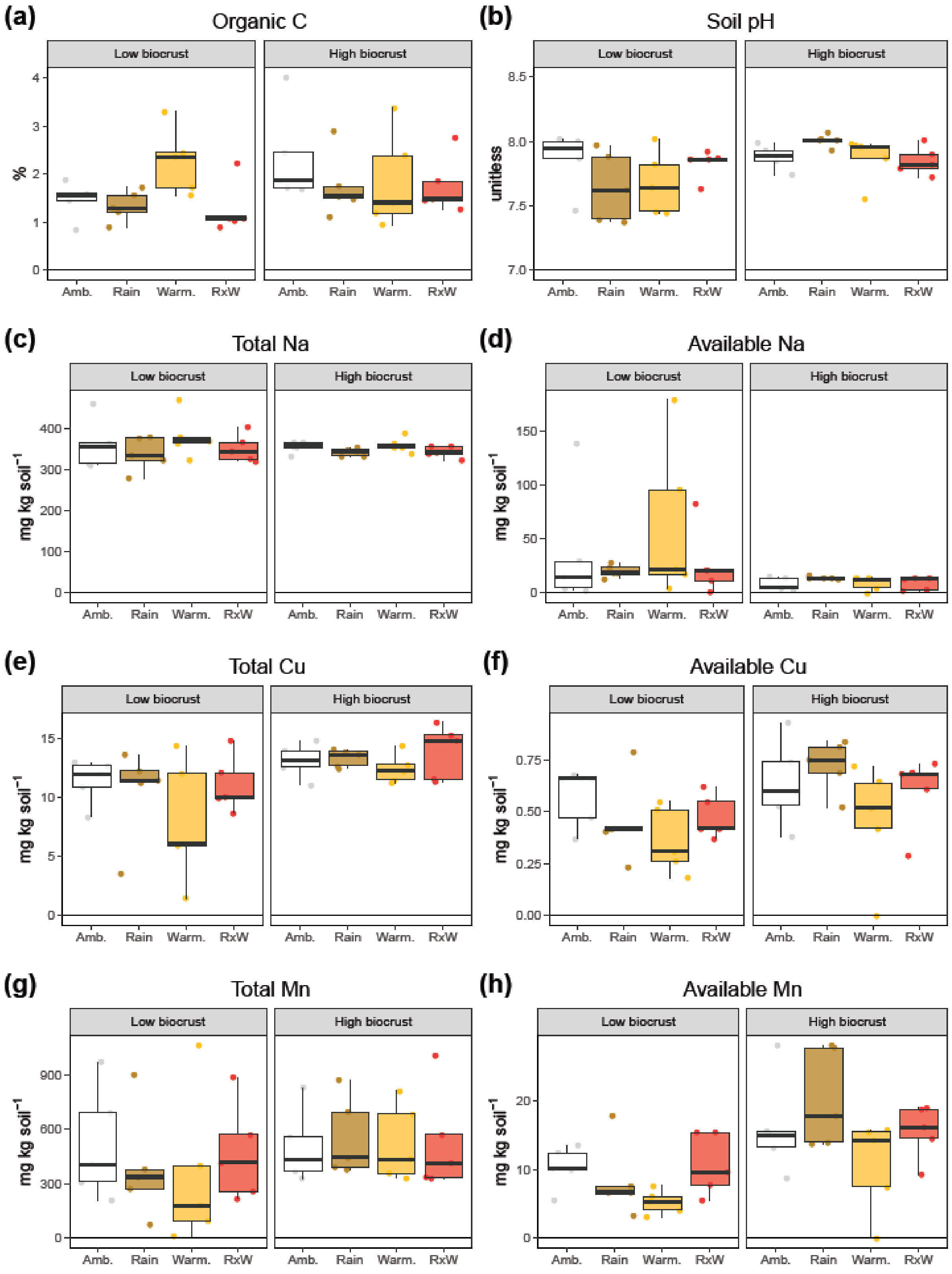

Supplementary Figure 4. *A priori* structural equation model used in this study. We included biocrust cover, warming, rain exclusion, soil pH and total metal concentration as predictors of DTPA-extractable metal. Also, the change metallic nutrient concentration (total and available 2017-2010) was tested in a second model. There are some differences between the *a priori* model and the final model structures owing to removal of paths with coefficients close to zero.

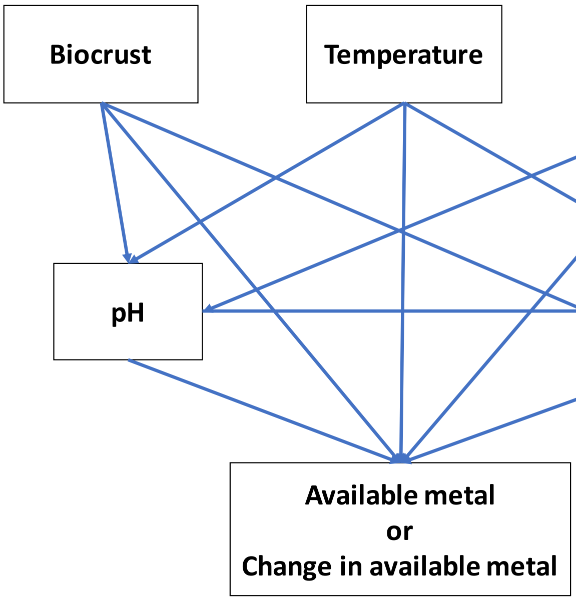

Supplementary Figure 5. Effects of biocrusts, warming, rainfall exclusion and soil pH on total and available Na, Cu and Mn in 2017, seven years after the beginning of the experiment. Numbers adjacent to arrows are standardized path coefficients (analogous to relative regression weights) and indicative of the effect of the relationship. Continuous arrows show positive and dashed arrows negative relationships, with arrow thicknesses proportional to the strength of the relationship. The proportion of variance explained (R^2) is shown besides each response variable in the model. Goodness-of-fit statistics are shown in the lower left corner as the χ^2. The *a-priori* model was refined by removing paths with coefficients close to zero (see the a-priori model in Supplementary Figure 4).

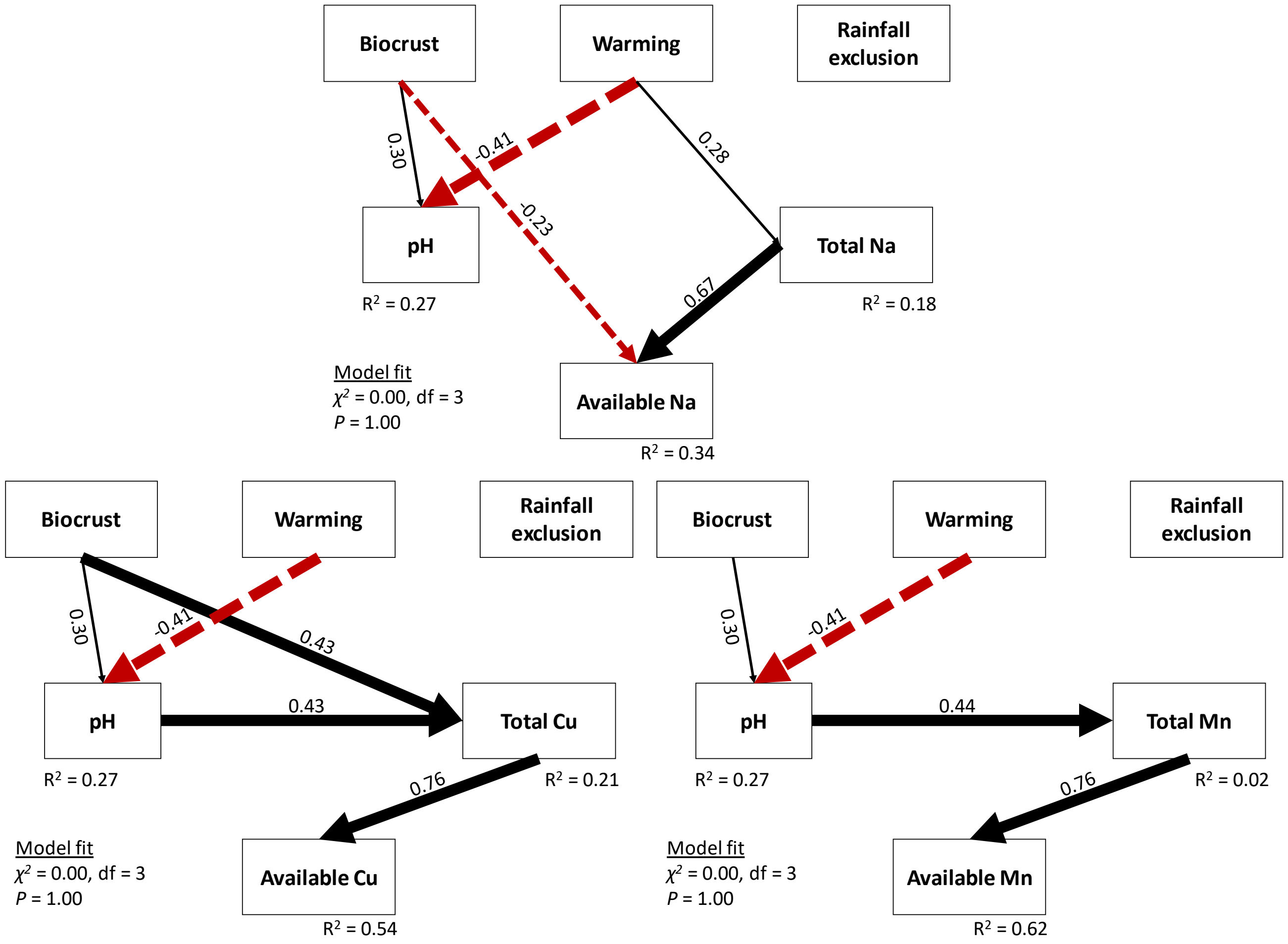

Supplementary Figure 6. Effects of biocrusts, warming, rainfall exclusion and soil pH on changes in total and available Na, Cu and Mn from 2010 to 2017. Numbers adjacent to arrows are standardized path coefficients (analogous to relative regression weights) and indicative of the effect of the relationship. Continuous arrows show positive and dashed arrows negative relationships, with arrow thicknesses proportional to the strength of the relationship. The proportion of variance explained (R^2) is shown besides each response variable in the model. Goodness-of-fit statistics are shown in the lower left corner as the χ^2. The *a-priori* model was refined by removing paths with coefficients close to zero (see the a-priori model in Supplementary Figure 4).

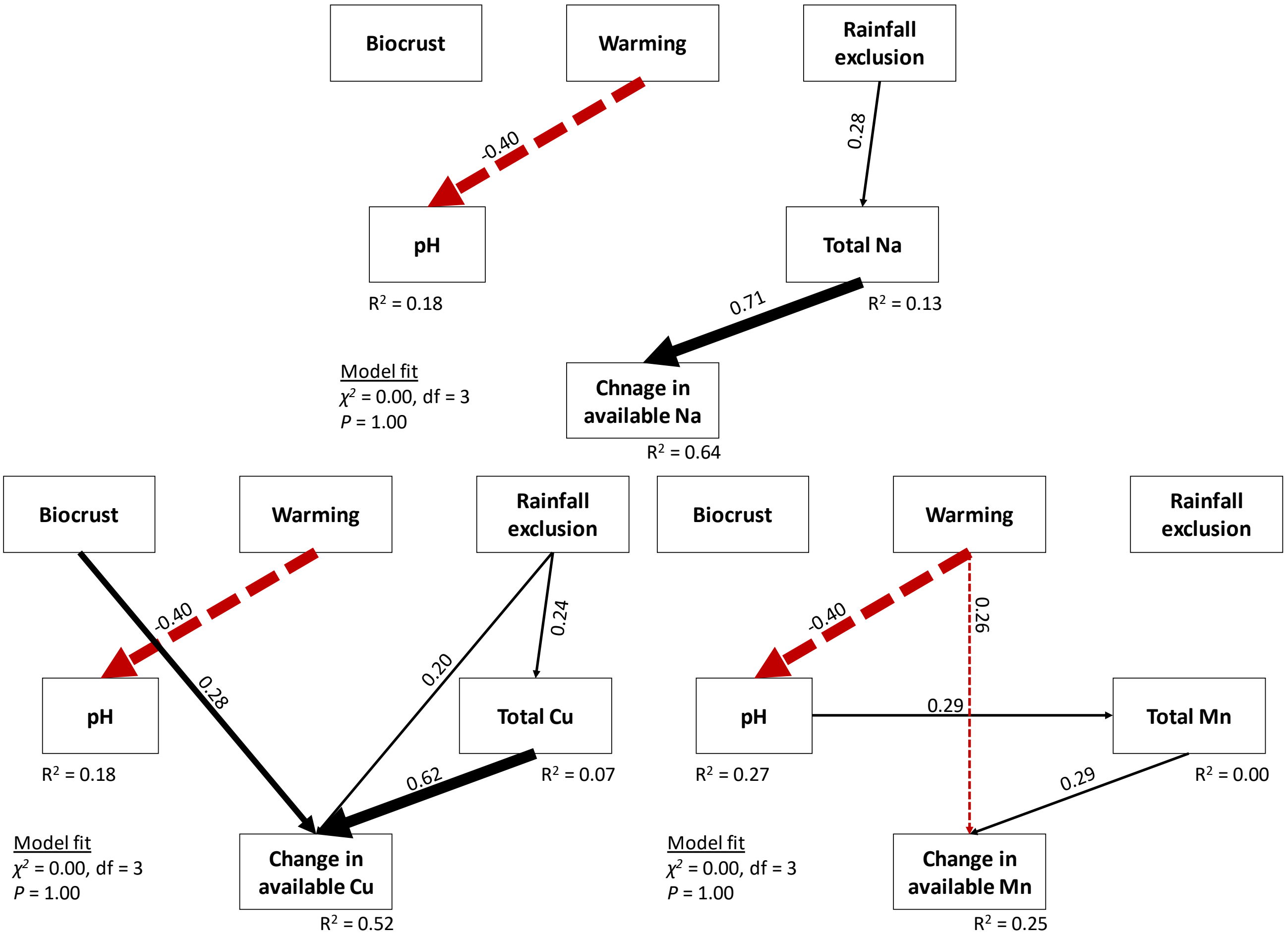

Supplementary Table 1. Results of ANOVA analyses showing the effects of biocrusts, rainfall exclusion and warming on total soil metallic nutrient contents, soil organic C content and soil pH seven years after the beginning of the experiment (2017). BC: biocrusts; RE: rainfall exclusion; W: warming. P values below 0.05 are in bold.

			**Total K**		**Total Na**		**Total Mg**		**Total Cu**		**Total Fe**		**Total Mn**		**Total Zn**		**Organic C**		**pH**	
	numdf	dendf	*F*	*P*	*F*	*P*	*F*	*P*	*F*	*P*	*F*	*P*	*F*	*P*	*F*	*P*	*F*	*P*	*F*	*P*
**BC**	1	32	6.78	**0.01**	0.99	0.33	17.21	**<0.01**	8.00	**0.01**	8.40	**0.01**	0.78	0.38	10.55	**<0.01**	4.16	**0.05**	4.17	**0.05**
**RE**	1	32	0.00	0.98	1.24	0.27	0.48	0.49	0.50	0.48	0.01	0.93	0.01	0.91	4.36	0.04	0.39	0.54	0.36	0.55
**W**	1	32	0.00	0.97	3.52	0.07	0.45	0.51	0.14	0.71	0.71	0.41	0.01	0.92	0.73	0.40	0.81	0.37	7.93	**0.01**
**BC:RE**	1	32	0.69	0.41	3.20	0.08	2.04	0.16	0.00	0.95	0.07	0.79	0.01	0.91	0.50	0.49	0.24	0.63	0.02	0.89
**BC:W**	1	32	0.58	0.45	1.40	0.25	0.05	0.82	0.02	0.90	0.00	0.96	0.20	0.66	0.27	0.61	0.44	0.51	0.04	0.84
**RE:W**	1	32	0.69	0.41	0.69	0.41	0.12	0.73	0.79	0.38	0.88	0.35	0.12	0.73	0.40	0.53	0.73	0.40	0.94	0.34
**BC:RE:W**	1	32	0.90	0.35	0.50	0.48	0.49	0.49	0.01	0.93	0.78	0.38	0.08	0.78	0.33	0.57	0.06	0.81	0.72	0.40

Supplementary Table 2. Results of ANOVA analyses showing the effects of biocrusts, rainfall exclusion and warming on total soil available metallic nutrients seven years after the beginning of the experiment (2017). BC: biocrusts; RE: rainfall exclusion; W: warming. P values below 0.05 are in bold.

			**Available Mg**		**Availalble K**		**Available Na**		**Available Cu**		**Available Fe**		**Available Mn**		**Available Zn**	
	numdf	dendf	*F*	*P*	*F*	*P*	*F*	*P*	*F*	*P*	*F*	*P*	*F*	*P*	*F*	*P*
**BC**	1	32	2.34	0.14	1.38	0.25	9.58	**<0.01**	4.54	**0.04**	10.14	**<0.01**	0.95	0.34	0.47	0.50
**RE**	1	32	0.58	0.45	0.90	0.35	4.19	**0.05**	1.09	0.31	0.24	0.63	0.46	0.50	15.57	**<0.01**
**W**	1	32	4.47	**0.04**	7.18	**0.01**	4.49	**0.04**	0.00	0.98	0.38	0.54	1.03	0.32	2.63	0.11
**BC:RE**	1	32	1.26	0.27	1.41	0.24	5.39	**0.03**	0.11	0.75	0.90	0.35	0.25	0.62	0.18	0.68
**BC:W**	1	32	1.05	0.31	3.15	**0.09**	1.15	0.29	0.01	0.92	0.00	0.98	0.82	0.37	1.18	0.29
**RE:W**	1	32	0.21	0.65	1.20	0.28	0.45	0.51	0.61	0.44	0.03	0.85	0.03	0.87	3.54	**0.07**
**BC:RE:W**	1	32	0.56	0.46	2.00	0.17	0.78	0.39	1.11	0.30	0.34	0.56	0.73	0.40	0.64	0.43

Supplementary Table 3. Results of PERMANOVA showing the effects of biocrusts, rainfall exclusion and warming on total and available macronutrients (Na, Mg and K) and micronutrients (including Fe, Zn, Cu and Mn) in soils at the beginning of the experiment (2010). Total: total; avail: available; macro: metallic macronutrients; micro: metallic micronutrients; BC: biocrusts; RE: rainfall exclusion; W: warming. P values below 0.05 are in bold.

			**totalmacro2010**		**totalmicro2010**		**availmacro2010**		**availmicro2010**	
	numdf	dendf	*F*	*P*	*F*	*P*	*F*	*P*	*F*	*P*
**BC**	1	32	4.17	**0.04**	6.74	**<0.01**	1.46	0.20	13.18	**<0.01**
**W**	1	32	2.42	0.15	0.36	0.83	0.85	0.45	2.05	0.09
**RE**	1	32	0.13	0.81	0.41	0.79	0.80	0.47	1.44	0.18
**BC:W**	1	32	1.68	0.21	0.29	0.92	0.48	0.77	0.41	0.86
**BC:RE**	1	32	1.39	0.24	0.44	0.79	0.34	0.89	0.40	0.86
**W:RE**	1	32	0.22	0.69	1.60	0.18	1.94	0.11	3.16	**0.04**
**BC:W:RE**	1	32	0.29	0.66	1.57	0.19	0.34	0.91	1.87	0.12

Supplementary Table 4. Results of PERMANOVA showing the effects of biocrusts, rainfall exclusion and warming on total and available macro (including Na, Mg and K) and micronutrients (including Fe, Zn, Cu and Mn) in soils in 2017 and on rate of change (Δ) between 2017 and 2010 of these elements. Total: total; avail: available; macro: metallic macronutrients; micro: metallic micronutrients; BC: biocrusts; RE: rainfall exclusion; W: warming. P values below 0.05 are in bold.

			**totalmacro2017**		**totalmicro2017**		**availmacro2017**		**availmicro2017**		**totalmacroΔ**		**totalmicroΔ**		**availmacroΔ**		**availmicroΔ**	
	numdf	dendf	*F*	*P*	*F*	*P*	*F*	*P*	*F*	*P*	*F*	*P*	*F*	*P*	*F*	*P*	*F*	*P*
**BC**	1	32	6.28	**0.02**	7.19	**<0.01**	6.40	**0.01**	1.77	0.17	1.13	0.36	1.99	0.15	2.42	0.08	1.02	0.39
**W**	1	32	0.07	0.87	0.59	0.49	7.17	**<0.01**	0.54	0.62	3.09	**0.03**	0.66	0.54	5.28	**<0.01**	1.82	0.14
**RE**	1	32	0.34	0.59	0.04	0.96	2.39	0.12	1.89	0.16	0.83	0.44	0.59	0.57	1.96	0.12	2.67	0.06
**BC:W**	1	32	0.03	0.96	-0.04	1.00	1.12	0.29	1.63	0.18	2.05	0.13	1.34	0.23	0.48	0.70	-0.39	1.00
**BC:W**	1	32	0.49	0.51	0.09	0.88	3.81	**0.04**	0.59	0.58	1.85	0.18	0.39	0.73	2.97	**0.04**	0.53	0.69
**W:RE**	1	32	0.13	0.78	1.20	0.27	0.23	0.84	0.36	0.73	0.88	0.43	0.81	0.45	0.96	0.39	1.00	0.40
**BC:W:RE**	1	32	0.06	0.87	1.01	0.37	0.90	0.37	0.70	0.50	0.10	0.90	0.48	0.67	0.81	0.49	0.55	0.68
