# Supplementary material for: Biocrusts buffer against the accumulation of soil metallic nutrients induced by warming and rainfall reduction
Source: Commun Biol. 2020 Jun 24;3:325. doi: 10.1038/s42003-020-1054-6 (PMC7314843; doi:10.1038/s42003-020-1054-6)
Supplement: Supplementary file 2 — Reporting Summary [file 42003_2020_1054_MOESM2_ESM.pdf]

nature research

Corresponding author(s): Eduardo Moreno Jiménez

Last updated by author(s): Jun 2, 2020

Reporting Summary

Nature Research wishes to improve the reproducibility of the work that we publish. This form provides structure for consistency and transparency in reporting. For further information on Nature Research policies, see our Editorial Policies and the Editorial Policy Checklist.

Statistics

For all statistical analyses, confirm that the following items are present in the figure legend, table legend, main text, or Methods section.

n/a	Confirmed	
☐	☒	The exact sample size (*n*) for each experimental group/condition, given as a discrete number and unit of measurement
☐	☒	A statement on whether measurements were taken from distinct samples or whether the same sample was measured repeatedly
☐	☒	The statistical test(s) used AND whether they are one- or two-sided<br>*Only common tests should be described solely by name; describe more complex techniques in the Methods section.*
☐	☒	A description of all covariates tested
☐	☒	A description of any assumptions or corrections, such as tests of normality and adjustment for multiple comparisons
☐	☒	A full description of the statistical parameters including central tendency (e.g. means) or other basic estimates (e.g. regression coefficient) AND variation (e.g. standard deviation) or associated estimates of uncertainty (e.g. confidence intervals)
☒	☐	For null hypothesis testing, the test statistic (e.g. *F*, *t*, *r*) with confidence intervals, effect sizes, degrees of freedom and *P* value noted<br>*Give P values as exact values whenever suitable.*
☒	☐	For Bayesian analysis, information on the choice of priors and Markov chain Monte Carlo settings
☒	☐	For hierarchical and complex designs, identification of the appropriate level for tests and full reporting of outcomes
☒	☐	Estimates of effect sizes (e.g. Cohen's *d*, Pearson's *r*), indicating how they were calculated

Our web collection on statistics for biologists contains articles on many of the points above.

Software and code

Policy information about availability of computer code

Data collection	No software was used to collect data. All data were produced in the experiment.
Data analysis	All the analyses were done in r software and associated codes

For manuscripts utilizing custom algorithms or software that are central to the research but not yet described in published literature, software must be made available to editors and reviewers. We strongly encourage code deposition in a community repository (e.g. GitHub). See the Nature Research guidelines for submitting code & software for further information.

Data

Policy information about availability of data

All manuscripts must include a data availability statement. This statement should provide the following information, where applicable:

- Accession codes, unique identifiers, or web links for publicly available datasets
- A list of figures that have associated raw data
- A description of any restrictions on data availability

All the data is available at: https://figshare.com/s/4e36e5b55a49bc038cc4

Field-specific reporting

Please select the one below that is the best fit for your research. If you are not sure, read the appropriate sections before making your selection.

☐ Life sciences ☐ Behavioural & social sciences ☒ Ecological, evolutionary & environmental sciences

For a reference copy of the document with all sections, see nature.com/documents/nr-reporting-summary-flat.pdf

Ecological, evolutionary & environmental sciences study design

All studies must disclose on these points even when the disclosure is negative.

Study description	It is a manipulative full factorial design
Research sample	Samples were taken from the soil surface, where biological activity is more intense, and the dried and sieved
Sampling strategy	We sampled five independet replicates
Data collection	Data were collected according to standard procedures, processed and analysed with appropriated analytical equipment and the results were processed and compiled in Excel files
Timing and spatial scale	Surface soils were sampled from the same area at the begining of the experiment and after seven years of treatment
Data exclusions	No data was excluded
Reproducibility	It is a manipuative experiment replicated in the field with independent adjacent plots
Randomization	Ramdomized blocks
Blinding	Samples were collected and numbered

Did the study involve field work? ☒ Yes ☐ No

Field work, collection and transport

Field conditions	Typical Mediterranean summer, 25-35 ºC
Location	Sorbas, Almería, South East Spain. Coordinates: 37°05′N–2°04′W; 397 m a.s.l.
Access & import/export	The researchers got permission from the landowner to set up and access the field experiment. No import/export of samples was needed
Disturbance	Sampling disturbed the plots. Since the experiment is still running, we minimized the impact by not sampling all the plots at the same time (e.g. changing sampling plots every sampling year)

Reporting for specific materials, systems and methods

We require information from authors about some types of materials, experimental systems and methods used in many studies. Here, indicate whether each material, system or method listed is relevant to your study. If you are not sure if a list item applies to your research, read the appropriate section before selecting a response.

Materials & experimental systems

n/a	Involved in the study
☒	☐ Antibodies
☒	☐ Eukaryotic cell lines
☒	☐ Palaeontology and archaeology
☒	☐ Animals and other organisms
☒	☐ Human research participants
☒	☐ Clinical data
☒	☐ Dual use research of concern

Methods

n/a	Involved in the study
☒	☐ ChIP-seq
☒	☐ Flow cytometry
☒	☐ MRI-based neuroimaging
